# ﻿Captive breeding, embryonic and larval development of *Ranitomeyavariabilis* (Zimmermann & Zimmermann, 1988), (Anura, Dendrobatidae)

**DOI:** 10.3897/zookeys.1172.98603

**Published:** 2023-07-26

**Authors:** Ruth Anastasia Regnet, Inna Rech, Dennis Rödder, Mirco Solé

**Affiliations:** 1 LIB, Museum Koenig, Leibniz Institute for the Analysis of Biodiversity Change, Adenauerallee 127, D-53113 Bonn, Germany Museum Koenig Bonn Germany; 2 Programa de Pós Graduação em Zoologia, Universidade Estadual de Santa Cruz, Rodovia Jorge Amado, Km 16, 45662-900 Salobrinho, Ilhéus, Bahia, Brazil Universidade Estadual de Santa Cruz Ilhéus Brazil

**Keywords:** Amphibians, ex situ, Gosner stages, metamorphosis, Neotropical, ontogeny, tadpoles

## Abstract

A solid basis to address the conservation challenges of amphibians requires an increased knowledge on their natural history and biology. Recent data on reproductive modes in amphibians suggest that they are much more complex and variable than previously thought but understudied. However, detailed information on the reproductive history is especially important to fill the current knowledge gaps. Following recent taxonomic changes in *Ranitomeyavariabilis*, information about captive-breeding management, image-based measurements of total length and surface area of the silhouette for individuals from embryonic to metamorphic development, and detailed larval staging for captive-bred specimens are provided from French Guiana. The development of *R.variabilis* from the stage eight (Gosner 1960) through metamorphosis took 79 to 91 days (*n* = 6) with a survival rate of 46%. The developmental stages largely matched those of the generalized staging system of Gosner (1960), with differences in the stages when labia and teeth differentiation and atrophy of the oral apparatus occurred. Compared with other studies the total length of *R.variabilis* tadpoles was greater at given stages than those of *R.variabilis* from a Peruvian population and those of the sister species, *R.amazonica*. Other studies concerning growth curves based on surface area data revealed that *R.variabilis* tadpoles at peak size were larger than those of *R.amazonica*, *R.imitator*, *R.reticulata*, *R.sirensis*, and *R.vanzolinii*, but smaller than *R.benedicta*. Our results represent the first embryonic and larval staging for *R.variabilis*, and detailed information is provided on their initial life phases. These data may facilitate the identification of *R.variabilis* tadpoles in the wild, as well helping to clarify the biogeographical distribution and taxonomic arrangement of the species. In addition, knowledge is added to the captive-breeding methodology for the species.

## ﻿Introduction

Amphibians have the emblematic status of being considered the most threatened group of vertebrates globally (Bishop et al. 2012). To provide a solid basis to address the conservation challenges of this group, greater knowledge is needed for amphibian natural history and biology. In particular, knowledge of reproductive biology is needed given that recent data on reproductive modes in amphibians suggest that they are much more complex and variable than previously thought (Nunes-de-Almeida et al. 2021).

To reduce the knowledge gap about amphibian reproductive biology, researchers are increasingly relying on captive-breeding programs as an ex-situ tool (e.g., Klein et al. 2020; Gray et al. 2021; Silla et al. 2021). The methodologies involved in captive breeding, however, still need to be further improved, and a greater understanding of their effects on amphibian individuals and populations is needed (McPhee 2004; Campbell-Palmer et al. 2006; Fraser 2008; Farquharson et al. 2021; Silla et al. 2021). The reputation of being easily adaptable to captive breeding is not always the reality and it is currently accompanied by a significant lack of knowledge about the impacts on ethology and physiology experienced by individuals in captivity (Campbell-Palmer et al. 2006). Moreover, when considering the high diversity of amphibian reproductive modes, the variety of different biological needs within the group is highlighted. Comparing with data obtained from natural environments, captive breeding allows to increase the understanding of the ontogeny of amphibians, as it greatly facilitates access to developing individuals and allows much easier collection of data in comparison to the often-difficult access to individuals in their natural environments (Gascon et al. 2007; Karlsdóttir et al. 2021; Linhoff et al. 2021).

As members of the family Dendrobatidae, the 16 species of the genus *Ranitomeya* Bauer, 1986 (Poison dart frogs), are characterized by their diminutive size (< 21 mm snout-vent length), almost smooth to slightly granular dorsal surface, bright aposematic coloration, and the first finger shorter than the second one (Daly et al. 1987; Vences et al. 2003; Brown et al. 2011; Muell et al. 2022). In addition, the genus is well known for its small anatomical (e.g., osteological) and morphological intraspecific variations, displays a great diversity of mating systems and coloration patterns, and often shows Müllerian mimicry among sympatric species (Muell et al. 2022). These species are also characterized by small clutch size and free-swimming tadpoles that are carried by adults to aquatic sites, where they continue their development (Brown et al. 2011; Sánchez 2013; Klein et al. 2020). Finding and correctly identifying the tadpoles of these species is often a difficult task in the natural environment. One approach is to observe the adults when they present parental care but they are also difficult to find because they are shy and small (Brown et al. 2011; Sánchez 2013).

Zimmermann’s Poison Frog *Ranitomeyavariabilis* (Zimmermann & Zimmermann, 1988) belongs to the *R.variabilis* species group, which is currently composed of two species, the Amazonian Poison Dart Frog (*R.amazonica* (Schulte, 1999)) and *R.variabilis*. Recently, the French Guiana and eastern Brazilian populations of *R.amazonica* were transferred to *R.variabilis* by Muell et al. (2022). Thus, currently, what is assumed as *R.variabilis* presents an eastern distribution in eastern French Guiana and the Pará region of Brazil, and a western distribution in the Andes Mountains of central Peru and western Ecuador (Muell et al. 2022). The current eastern and western *R.variabilis* populations present a disjunct distribution with a huge distance between them and their biogeographical distribution and taxonomic arrangement need further investigation. This could help to assess its conservation status, at it is currently listed as data deficient according to the Red List of the International Union for Conservation of Nature (IUCN 2021).

*Ranitomeyavariabilis* is semi-arboreal and inhabits primary and secondary rainforest (Lescure and Bechter 1982; Brown et al. 2011). The species can be found usually in ground vegetation or in vertical-growing plants as well as in leaf litter on the banks of streams and small rivers (Lescure and Bechter 1982). The species presents diurnal habits and a highly complex social and reproductive behavior, with a promiscuous mating strategy (Brown et al. 2011; Muell et al. 2022). Clutches are composed of 1–6 eggs, which are deposited adhered on the phytotelmata slightly over the edge of the water (Poelman and Dicke 2007). Males provide parental care that includes transport of one or two tadpoles at a time and posterior single deposition in phytotelmata to complete their development (Lötters et al. 2007; Poelman and Dicke 2007; Brown et al. 2011). Tadpoles have a cannibalistic strategy by predating on conspecific eggs when optimal reproductive conditions in the phytotelma decline. This strategy has been described for the species under the synonym *Dendrobatesventrimaculatus* Shreve, 1935 (Poelman and Dicke 2007), which was later related to *R.amazonica* (Brown et al. 2011). Observations on the reproductive behavior of *R.variabilis* during captive breeding were precisely described under the specific name *D.quinquevittatus* Steindachner, 1864 (Lescure and Bechter 1982), which was later related to *R.amazonica* (Brown et al. 2011). This description of the reproductive behavior includes details on courtship ritual, emission of spermatozoa, spawning, fertilization, and behavior of adult males during the hatching of tadpoles. Lescure and Bechter (1982) also described the tadpole mouthparts, and external morphology of a tadpole at stage 27 and at stage 38 (Gosner 1960), for specimens from Cacao and Montsinery, French Guiana. Furthermore, Masche et al. (2010) presented a second description of *R.variabilis* tadpoles, based on captive-bred specimens from the Tarapoto region in Peru.

However, following the current classification of *R.variabilis*, it is evident that the biogeographic and taxonomic arrangement of this species is still unclear, as shown by the distance between eastern and western populations and the variety of morphotypes (Muell et al. 2022). The aim of the present study was to describe in detail the development from embryo to metamorph of *R.variabilis*, based on captive-reared specimens from a population of French Guiana. We confirmed the species identity by DNA barcoding. We provide the following novel information for the embryos and larvae of the species: (1) captive-breeding management for this species; (2) image-based measurements of total length (TL) and surface area of the silhouette of individuals from embryonic to larval development; and (3) detailed staging from early-stage embryo to metamorph, including photographs. Furthermore, we compare the new data for *R.variabilis* early life stages with developmental data of other *Ranitomeya* species, as well as with tadpoles of the Peruvian *R.variabilis* population.

## ﻿Materials and methods

### ﻿Specimen acquisition and species identity

We acquired adult specimens of *R.variabilis* from the pet trade, which we kept in customized terraria at the Leibniz-Institut zur Analyse des Biodiversitätswandels (LIB), Zoologisches Forschungsmuseum Alexander Koenig, Bonn, Germany. According to the traders, the specimens were captive bred F_2_ from specimens exported from French Guiana, which had been acquired from Peruvian Frog Import (Doetinchem, Netherlands). We kept *Ranitomeyavariabilis* individuals at LIB Bonn without contact with other species of the genus, preventing hybridization between species. We confirmed the correct taxonomy of the species by comparing the external morphology with the respective original descriptions and by DNA barcoding. For the latter, we extracted DNA from two embryos that were not used in the measurements described below and sequenced a fragment of the mitochondrial 12s rRNA (see Koch et al. 2013 for laboratory procedure). We compared the obtained sequences (GenBank accession numbers OQ547269–OQ547270) to 168 sequences of *Ranitomeya* species available on GenBank.

### ﻿Captive management and breeding

We followed the captive-breeding protocol established for other *Ranitomeya* species by Klein et al. (2020). We housed our specimens in a terrarium of (100 × 40 × 60 [length, width, height]), equipped with a 5-cm filter-mat on the bottom, and the rear and the sides covered with Hygrolon (Dusk Tropic, Saltsjö-Boo, Sweden). This is a non-decomposable, highly hygroscopic synthetic tissue, which guarantees high air humidity in the terrarium (Behr and Rödder 2018; Galunder and Rödder 2018). We provided artificial daylight daily from 0800 to 2000 with an LED light (Solar Stinger, 1100 Sunstrip Dimmable Driver, 25 W; 2250 lm, Econlux GmbH, Cologne, Germany). In addition, we included an irrigation system, a small water part (10 × 100 cm) with a drain in a sieve form, located across the front of the terrarium, and a misting system. We activated the misting system three times a day, for twelve alternating intervals of each 10 s spraying followed by 10 s pause, for a total of 120 s of spray. We measured average air and water temperature using a digital thermometer (Pearl GmbH, Buggingen, Germany); both fluctuated between 22 and 26 °C.

To provide opportunities of shelter, and also to ensure a vertical structure and potential breeding sites, we densely planted the terrarium with Polka Dot Begonia (*Begoniamaculata*), shingling vine (*Marcgravia* sp.), Silver Vine (*Scindapsusaureus*), and Flaming Sword Bromeliad (*Vrieseasplendens*), providing important resources as natural phytotelmata (e.g., Lescure and Bechter 1982; Klein et al. 2020). Additionally, we equipped the terrarium with cylindrical photographic film containers (33 mm diameter; 54 mm deep), oak (*Quercus* sp.) leaf litter sterilized by cooking, roots, and stones. We placed film containers on the terrarium side walls next to a plant and in a vertical position, slightly inclined to keep a small amount of water at the bottom and to provide additional artificial phytotelmata. We fed the frogs with a diet of collembolans (springtails), fruit flies (*Drosophila* sp.), or small house crickets (*Acheta* sp.) every 2–3 d, enriched with vitamins and minerals (Herpetal Mineral+Vitamin D3, Korvimin ZVT+Reptil; Keweloh Animal Health GmbH & Co KG, Neuenkirchen, Germany).

To obtain ontogenetic development data, we removed clutches from the phytotelmata with the aid of pipettes and kept each embryo separately in a properly labelled petri dish. We placed these in an environmental test chamber (MLR-352H-PE; Panasonic Biomedical Sales Europe BV, Etten-Leur, Netherlands) to ensure stable conditions of 80% relative humidity, 24 °C, and a 12-h photoperiod from 0800 to 2000 hrs. Every 2 d we wetted the embryos with reverse-osmosis water, according to the captive-breeding protocol for other *Ranitomeya* species (Klein et al. 2020). After hatching, we kept each specimen separately in the environmental chamber in small translucent plastic containers (10 × 10 × 10 cm) filled with fresh osmosis water, a piece of Tropical almond tree (*Terminaliacatappa*) leaf, as well as a stem of Water aspidistra (*Anubiasbarteri*) and Spiky moss (*Taxiphyllum* sp.). The setup was prepared 4 d before the individual was introduced to allow for stabilization of the microenvironment. Every 2–3 d we exchanged 2/3 of the water to preserve the favorable environment for the tadpole. We fed the larvae every 2–3 d with Repashy Superfoods, Savory Stew Omnivore Gel Premix (Repashy Ventures-Specialty Pet Products, Oceanside, California, USA) ad libitum. To avoid the accumulation of food remains, we cleaned the bottom of the container during the water changes, but we retained the biofilm that was naturally formed on the walls, allowing the tadpoles to graze on these biofilms and algae.

When the hind limbs were fully developed and before the forelimbs emerged, at development stage 40 (Gosner 1960) we placed a small piece of cork tile on the water surface to provide a small land area for metamorphosing individuals. During the latest steps of metamorphosis, when the tail was resorbed at development stages 45–46, we transferred individuals to a small terrarium (40 × 50 × 40 cm [length, width, height]) with the same environmental conditions as the terraria of the adults and equipped in a similar way with plants, refuges, and also a small water body. From this moment on, we fed the fully metamorphosed frogs with a diet of collembolans and after approximately two weeks also with small fruit flies and small crickets.

### ﻿Growth and development data and evaluation

To classify the developmental stages of individuals, we used the staging system for anuran embryo and larvae by Gosner (1960) and adapted into the four major developmental categories, by McDiarmid and Altig (1999): embryo (Gosner stages 1–19), hatchling (stages 20–25), larva (stages 26–41), and metamorph (stages 42–46). In the text, we refer to tadpoles as the free-swimming phase of the individuals, i.e., larva and metamorph stages. To document growth and development of the specimens, we took photographs with the aid of a stereomicroscope and its integrated eyepiece (Stemi 2000 C; Carl Zeiss Microscopy GmbH, Jena, Germany) with attached camera (EOS 600D; Canon Deutschland GMBH, Krefeld, Germany) due to the small size of the individuals up to stage 25. For the photographs from stage 26 until the completion of metamorphosis, we used the same camera, but mounted it on a table-top tripod at a fixed distance to the subject. To analyze growth of the specimens, we used the software SAISAQ as described in Kurth et al. (2014). We processed the photographs from developmental stages 8–43 through the open-source platform R, version 4.1.0 (R Core Team 2021). The software calculates the surface area of the silhouette from each specimen, based on photographs, and generates a growth curve throughout development.

We took photographs of the individuals twice a week for the SAISAQ methodology, following Klein et al. (2020). Where the specimens individually deposited on a petri dish, were illuminated from below, and the resulted photo are a silhouette of the individual. After each specimen photo, a standard graph paper photo was also taken in order to obtain the correct measurements of the individuals. To document the ontogeny, we illuminated the individuals from above and photographed them dorsally and ventrally. To detect as many developmental stages as possible, and due of the rapid ontogenetic changes in the embryonic stages, we photographed embryos every day. We photographed individuals in subsequent stages twice a week. The average growth across all specimens was estimated using a local polynomial regression (Cleveland et al. 1992). In this method as implemented in the R package stats (R Core Team 2021), model fitting is computed locally: the fit at a point x is calculated using points in a neighborhood of x, weighted by their distance from x. As size of the neighborhood, we applied the default settings of 0.75. As error estimate we computed the 95% confidence interval of the Loess function. We obtained further image-based measurements, such as embryo size measurements, TL, caudal length, using the software ImageJ v. 1.53 (Schneider et al. 2012), calibrated in millimeters by photographs of graph paper taken with each individual. We present measurements data as mean ± standard deviation (*SD*) and range for each development stage.

## ﻿Results

### ﻿Species identity

We found that the sequences of the two barcoded specimens were identical to a sample from Kaw, French Guiana (GenBank accession number DQ163087). Furthermore, our specimens fitted the color pattern featured in the Kaw population (see Brown et al. 2011). This population originally had been identified as *R.variabilis*, but was later revised as *R.amazonica* (Brown et al. 2011). Subsequently, the French Guiana populations of *R.amazonica* were transferred to *R.variabilis* (Muell et al. 2022).

### ﻿Captive breeding observations

Prior to oviposition, we observed that the nine *R.variabilis* adults (5 females, 4 males) had gathered in pairs or in small groups of up to five individuals, with males constantly vocalizing. When they had gathered in groups, usually more than one male vocalized at the same time. Calling males did not show aggressive behavior towards each other. Apparently, oviposition occurs in the early hours of the day, since this species has diurnal habits and no new spawning was observed during the day; new spawn was always laid during the morning period (0730–0900 am). The females placed the eggs in clutches of 4–6 dark grey eggs, individually wrapped in a gelatinous capsule. We detected embryos on the leaves of the bromeliads, slightly over the edge of the water, but not in the water. The plastic film containers were less frequently used for egg deposition than bromeliads leaves. When deposited in plastic containers, females placed the clutches at the bottom of the container, directly below the water surface. The clutches in containers, however, were disturbed by stepping by the adults, which sometimes even defecated inside the containers on top of the spawn. This apparently reduced their development success, compared to the embryos placed on bromeliads. We observed no clutches in other parts of the terrarium and we did not observe a clear temporal frequency of egg deposition.

When left in the terrarium, clutches with embryos enveloped in colorless albumin, deposited on bromeliads leaves, often slipped into the water before the tadpoles hatched. Adults carried on their backs some of the tadpoles that hatched on bromeliads to the water body inside the terrarium or to another bromeliad. However, some hatched tadpoles ended up staying in the bromeliad phytotelmata of the deposition site. The frogs preferred to deposit the tadpoles in the bromeliad axis or in smaller areas of the puddle, such as a small puddle formed between aquatic plants or an area surrounded by rocks within the puddle. Frequently, we observed adults carrying one tadpole at a time. In the bromeliad axils, the frogs deposited tadpoles only individually, while in the puddle, they deposited up to three tadpoles of different developmental stages together. We did not observe predation or conflict between smaller and larger tadpoles deposited together. There was no noticeable parental care of the tadpoles after being transported to the water body, such as feeding of embryos with trophic eggs.

### ﻿Growth and development

We obtained 13 embryos of *R.variabilis*, from four separate spawnings produced between January and February 2021. Our observations began during the early embryonal phase (stage 8), because we were not able to detect eggs/embryos in the first seven developmental stages. We document below the development of seven of these individuals in detail, seven through the embryo and hatchling stages and six until metamorphosis. The individual that did not reach metamorphosis showed slower development and reduced size in the larval and metamorph stages in comparison to the other six tadpoles. For example, when this individual reached stage 38 it had a TL of 24 mm compared to the 29–33 mm TL of the other six specimens at the same stage. Furthermore, when it reached stage 38 the other six individuals had already reached stages 42–43. This individual needed 117 days to fully develop its hind limbs at stage 41 and TL = 24 mm, but it died before the forelimbs emerged.

Six of the 13 individuals did not develop well and died before completing metamorphosis. Thus, they were not included in the detailed descriptions of ontogeny below. Four of these individuals, in approximate developmental stages 37–40, presented malformations of the limbs, such as visually thinner limbs compared to apparently healthy tadpoles, as well as missing fingers. The other two individuals died in the embryonic stage. Survival to metamorphosis among the 13 individuals was thus 46%.

**Figure 1.**
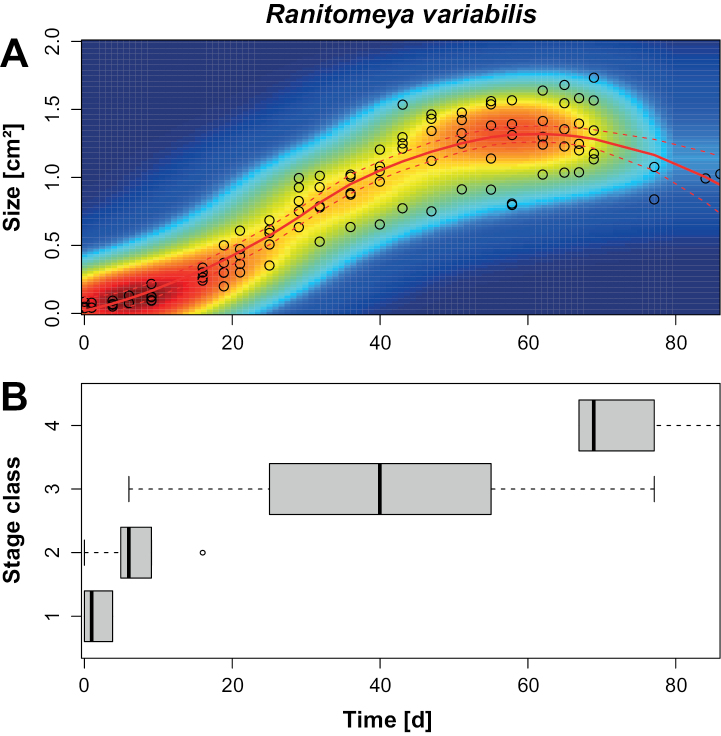
**A** size and growth curve (i.e., size across time; red continuous line) of *Ranitomeyavariabilis* from French Guiana, from embryo (Gosner [1960] stage 8) starting on day 0 to metamorphic stage 43 (*n* = 6). Size is represented as the surface area of silhouette of individual. Time represents days since beginning of observations (stage 8). Each circle represents one individual at the given time. The color range indicates the density of observations, from blue (no observations) to red (highest density). The growth curve is represented by the Loess function. The dotted lines display the 95% confidence interval of the Loess function **B** boxplot, stages: 1 = Embryo; 2 = Hatchling; 3 = Larvae, and 4 = Metamorph.

For the six individuals that completed metamorphosis, development from stage 8 to complete metamorphosis (stage 46) took between 79 and 91 days (mean = 81.8 d; *SD* = 4.6 d; Table 1). For five of these individuals, each developmental stage was reached at nearly the same time, and complete metamorphosis took 79–80 days. The two body size measurements, TL and surface area of the silhouette, were strongly correlated (simple linear regression based on mean values during each stage): Surface Area (cm^2^) = 1.0538 TL (mm) + -0.1527 (R^2^ = 0.9441). Both total length and surface area of the silhouette increased throughout most of development, then peaked and declined (Table 1; Fig. 1). Total length peaked at stage 40 (58–65 d), shortly before metamorphosis began at stage 42. The measured surface area peaked during metamorphosis at stage 43 (69–72 d). The fitted curve for surface area showed an earlier peak at ca. 63 days (Fig. 1).

**Table 1.** Size and developmental time for *Ranitomeyavariabilis* from French Guiana. Abbreviations: n = total of individuals; Stg = Gosner stage of development; Time = development time, i.e., days since beginning of observations (stage 8) when the given stage was reached; TL = Total Length; SA = Surface area of silhouette of individual; SD = standard deviation.

Number	Stg	Time	TL (mm)		SA (cm^2^)	
		(d)	mean±SD	range	mean±SD	Range
Embryo n=7	8	0	2.20±0.00	2.20	0.05±0.02	0.04–0.08
	11	1	2.50±0.00	2.50	0.05±0.02	0.04–0.08
	19	4	4.70±0.00	4.70	0.07±0.02	0.05–0.09
Hatchling n=7	20	5	7.66±0.08	7.50–7.70	–	–
	21	6	8.44±0.12	8.20–8.50	0.08±0.04	0.04–0.13
	24	9–10	10.32±0.85	9.50–11.80	0.12±0.07	0.06–0.21
	25	10–11	14.61±1.70	11.20–18.00	0.27±0.15	0.08–0.61
Larva n=6	26	21–25	17.78±0.27	17.40–18.00	0.50±0.30	0.32–0.80
	27	21–26	19.10±0.37	18.50–19.50	0.63±0.25	0.35–1.01
	28	25–28	21.70±0.75	21.00–23.00	0.76±0.26	0.51–1.02
	29	29	23.52±1.34	21.50–25.00	0.89±0.15	0.63–1.08
	30	31	24.66±1.65	22.50-27.00	–	–
	31	32–33	25.64±1.57	23.80–28.00	0.91±0.37	0.52–1.53
	32	36	26.84±1.55	25.00–28.70	0.95±0.23	0.63–1.34
	34	40–43	28.68±1.76	26.00–31.00	1.09±0.25	0.65–1.32
	35	43–44	28.70±1.98	26.50–31.00	1.17±0.31	0.77–1.54
	36	44–47	29.88±2.01	27.00–32.00	1.14±0.29	0.75–1.43
	37	47–51	30.20±2.08	27.50–32.50	1.35±0.22	0.91–1.58
	38	51–56	31.20±1.83	29.00–33.00	1.34±0.29	0.91–1.47
	39	55–58	31.60±1.85	29.00–34.00	1.13±0.43	0.81–1.56
	40	58–65	32.00±1.91	29.00–34.00	1.31±0.22	1.02–1.73
	41	58–68	31.70±1.94	29.00–34.00	1.34±0.23	1.03–1.64
Metamorph n=6	42	67–70	31.00±1.79	29.00–33.00	1.23±0.15	1.04–1.40
	43	69–72	28.10±0.92	26.50–29.00	1.35±0.18	1.13–1.56
	46	79–91	11.50±0.40	11.51	–	–

### ﻿Growth and development: embryonic stages (stages 8–19)

**Figure 2.**
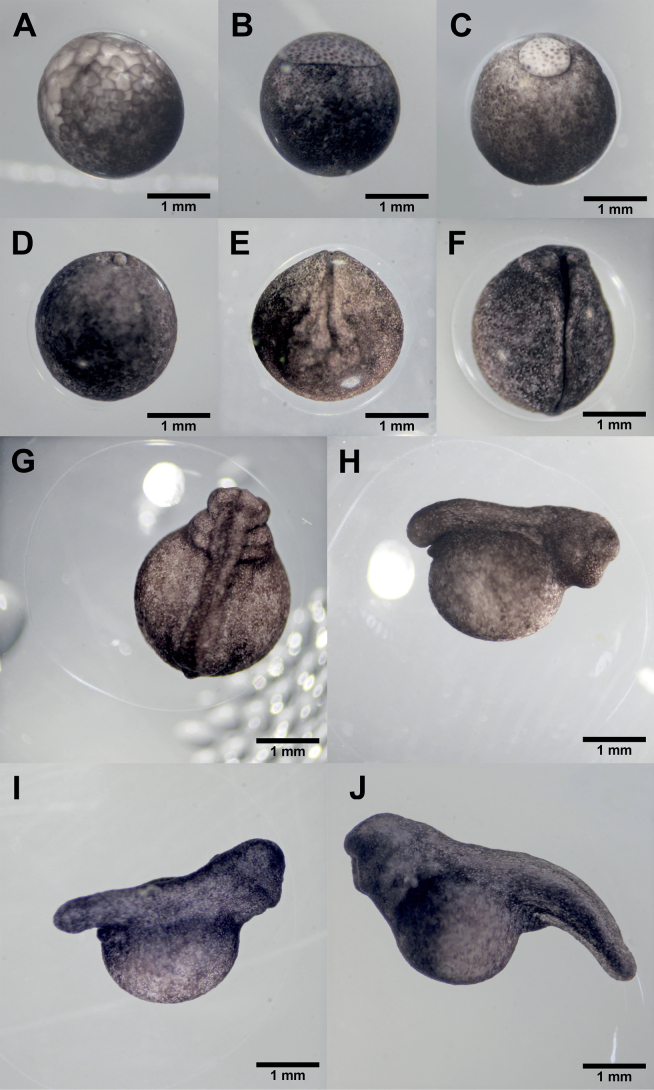
Embryonic development of *Ranitomeyavariabilis* from French Guiana at Gosner (1960) stages 9–19 **A** stage 9 **B** stage 10 **C** stage 11 **D** stage 12 **E** stage 14 **F** stage 15 **G** stage 16 **H** stage 17 **I** stage 18 **J** stage 19.

Oviposition apparently occurred in the early hours of the day, given that we always located new spawn during the morning period (0730–0900) and never later during the day. We thus estimated that fertilization occurred a few hours prior to the start of our observations at stage 8 (Table 1). At stage 8 (mid cleavage/morula), the embryos were encompassed by a capsule and gelatinous layer and were pigmented dark-grey on the upper part and pale beige on the part facing the substrate where the spawn was adhered, and the mean capsule diameter was 2.2 mm. In stage 9, corresponding to the late cleavage/blastula, the pigmented area at the animal pole (dark grey coloration) extended over the vegetal pole (pale grey coloration; Fig. 2A). One day after the start of our observations the embryos had reached stage 10 and gastrulation had begun (Fig. 2B). At stage 11, the animal pole surface extended over the vegetal pole, reducing the exposed area of the vegetal pole; the yolk plug had appeared and was easily discernible by its pale gray coloration (Fig. 2C). Two days after the beginning of our observations the embryo reached stage 12 and the yolk plug appeared as a small pale gray colored button (Fig. 2D). In stage 13, the neural plate became visible on the dorsal area; subsequently it became flat, and the embryo became slightly elongated. In stage 14 the neural folds were evident (Fig. 2E).

After three days, the embryo reached stage 15, the elongation was visible, and the structures to give rise to the anteroposterior regions could be identified (Fig. 2F). At stage 16, the neural fold closed and formed the neural tube; the head, gill plates, and the tail bud started to develop and were distinguishable; and the diameter of the capsule enclosing the embryo had increased, reaching a mean of 4.3 mm (Fig. 2G, H). The embryo reached stage 18, at which time a large yolk sack was discernible in the ventral region and the tail bud had elongated further (Fig. 2I). The first muscular response was noticeable through spontaneous wriggling. The olfactory pit developed at the ventral part of the head, and gill plates protruded. At four days, at stage 19, the embryonic body formed a larval shape (Fig. 2J). Thus, the head and tail regions were strongly evident, and the gill buds began to develop and could be detected at the intersection of the yolk sac and the head. A heartbeat was observed below the gills.

### ﻿Growth and development: hatchling stages (stages 20–25)

**Figure 3.**
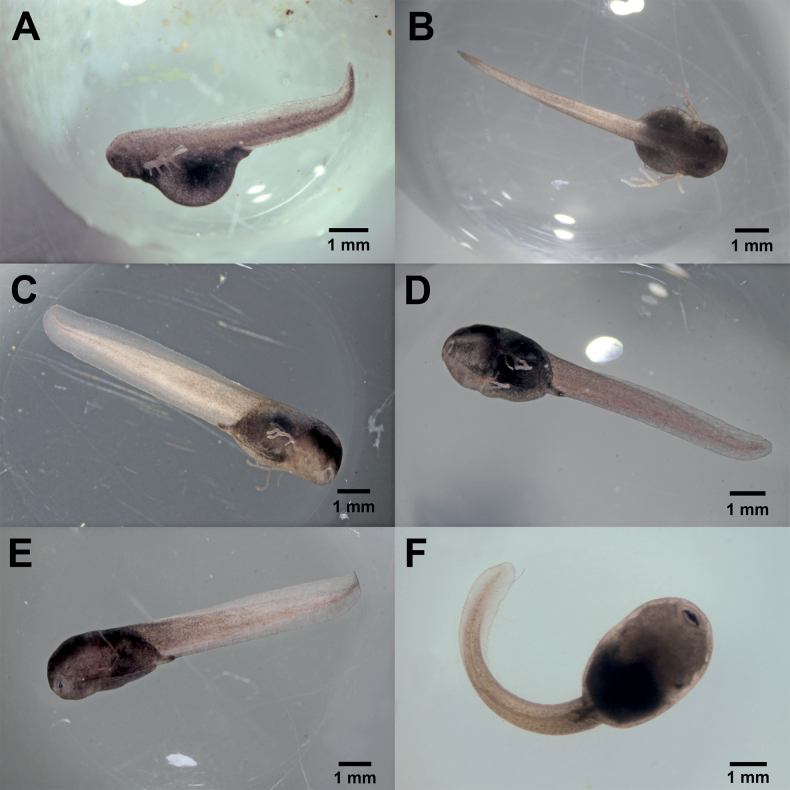
Hatchling development of *Ranitomeyavariabilis* from French Guiana at Gosner (1960) stages 20–25 **A** stage 20 **B** stage 21 **C** stage 22 **D** stage 23 **E** stage 24 **F** stage 25.

Stages 20 and 25 were reached at 5 and 10 days, respectively (Table 1). At stage 20, the tail had elongated and the upper and lower caudal fins together with the myosepta became visible (Fig. 3A). Moreover, the overall body, as well as the external gills increased in size, and gill circulation became evident at the same time as the yolk sack started to atrophy. At stage 21, the tail continued to lengthen, the eyes became visible through the transparent cornea, the mouth opened ventrally, and the external gills were fully developed. The hatchling exhibited rapid movements inside the gelatinous layer in response to external disturbances, such as when handling the petri dish.

The hatchling reached stage 22 after seven days of observations; at this time the nares were discernible and the dorsal and ventral caudal fins became higher and transparent (Fig. 3B, C). The diameter of the capsule enclosing the embryo also increased (mean = 10 mm). At stage 23, spotted beige dots on the tail fins became visible (Fig. 3D). The operculum covered the external gill bases which began to be resorbed, the yolk sack was almost completely atrophied, and the labial ridge and labial teeth were slightly visible. In a development time of 9–10 days after the beginning of observations, all hatchlings had reached stage 24 (Fig. 3E), the typical tadpole mouthparts were completely formed, and labial papillae and teeth rows were easily distinguishable (Fig. 3E). Due to the increase in size, the hatchlings were slightly curved inside the gelatinous layer. The operculum folds covered the external gills and were closed on the right side, while on the left side; the external gills remained uncovered with different degrees of atrophy among the individuals. Stage 25 was reached after 10–11 days from onset of observation and extended until day 21. In this stage the left external gill atrophied, and the sinistral spiracle and operculum were formed (Fig. 3F). The tail length reached its maximum size for this development category (mean = 7 mm) and the hatchling was very curved within the gelatinous layer. At 16 days after the start of observations, all hatchlings had emerged from the capsule and gelatinous layer and swam free in the water.

### ﻿Growth and development: larval stages (stages 26–41)

**Figure 4.**
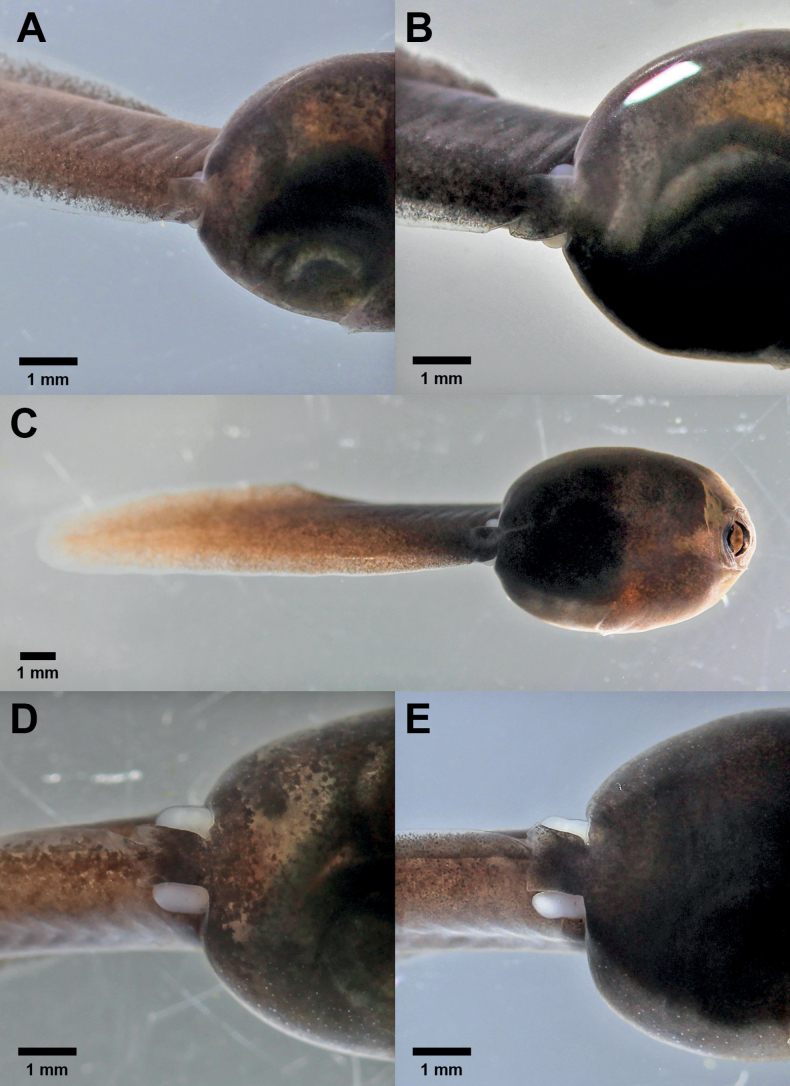
Larval development of *Ranitomeyavariabilis* from French Guiana at Gosner (1960) stages 26–30 **A** stage 26 **B** stage 27 **C** stage 28 **D** stage 29 **E** stage 30.

**Figure 5.**
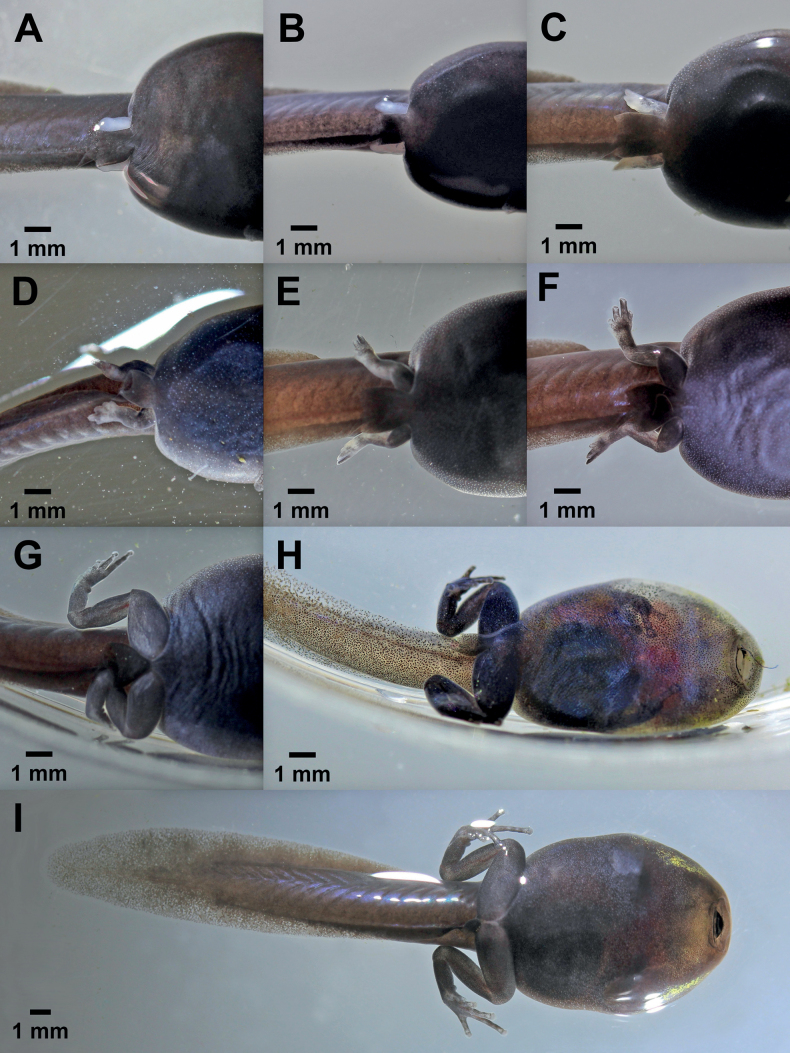
Larval development of *Ranitomeyavariabilis* from French Guiana at Gosner (1960) stages 31–41 **A** stage 31 **B** stage 32 **C** stage 34 **D** stage 35 **E** stage 36 **F** stage 37 **G** stage 39 **H** stage 40 **I** stage 41.

Stage 26 was reached at 21–25 days and stage 41 was reached at 58–68 days (Table 1). Between stage 25 (hatchling) and stage 26 (larva) the tadpoles increased considerably in size; during this time, they grew from mean = 14.6 mm (stage 25) to mean = 17.8 mm (stage 26; Table 1). At stage 26 the hind limb buds became visible for the first time (Fig. 4A); the bud length was shorter than half of its diameter. In three of the six larvae, we observed that the right bud appeared 1 day before the left one. The development of the hind limb buds, from stage 26 until stage 30, when the bud length had reached twice its diameter, took 10–11 days (Fig. 4A–E). At the same time, the tadpole increased in size from a mean = 17.8 mm (stage 26) to mean = 24.0 mm (stage 30; Table 1). From stage 31 onwards, the differentiation of the toes in the hind limb bud became apparent (Fig. 5A–I). The first melanophore spots appeared on the hind limbs at stage 34 (Fig. 5C). The first signs of the dorsal color pattern characteristic of the adult frogs also appeared at this stage.

At stage 37, all toes became separated and started to elongate (Fig. 5F). In the subsequent stages, the hind limbs continued to develop and increase in size; the metatarsal tubercles and subarticular toe patches were present at stage 38 and 39 (Fig. 5G). In a development time of 58–65 days from the beginning of observations, all tadpoles had reached stage 40 and their maximum mean length of 32.0 mm (Table 1). The forelimbs, still inside the body, were visible through the skin. The ventral tube was still present and two of the six tadpoles, stage 40, lost the buccal apparatus (Fig. 5H). Stage 41 was reached after 58–68 days (Table 1) and the development of the hind limbs was completed (Fig. 5I). The forelimbs were evident as elevations next to the operculum. Oral apparatus atrophy and ventral tube absorption took place in all specimens before reaching stage 42.

### ﻿Growth and development: metamorphic stages (stages 42–46)

**Figure 6.**
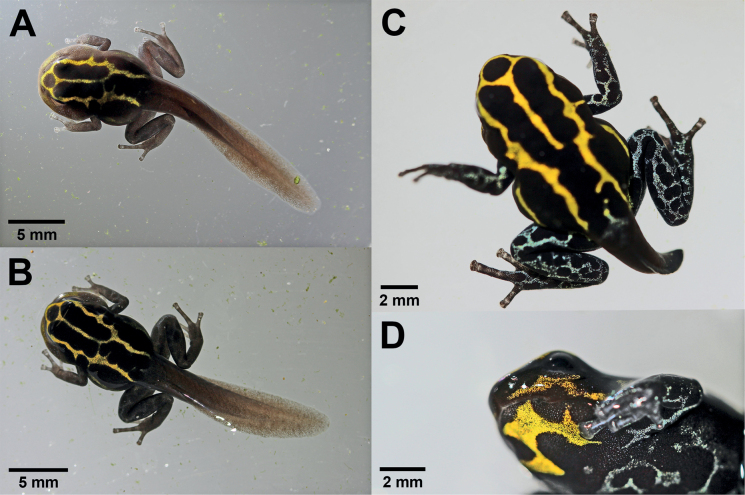
Metamorph development of *Ranitomeyavariabilis* from French Guiana at Gosner (1960) stages 42–44. **A** stage 42 **B** stage 43 **C** stage 44 **D** stage 44 in lateral view. In **D** the mouth angle is located beneath the eyes, and the gular region and part of the venter show typical adult coloration.

Stage 42 was reached at 67–70 days and stage 46 was reached at 79–91 days (Table 1). Five of the individuals reached stage 42 after 67 days from the start of reporting, only one individual took up to 70 days to reach this stage. At stage 42, while the forelimbs emerged and the tail began to be resorbed, changes in the mouth shape also began (Fig. 6A). In lateral view the mouth angle was already located anterior to the nostril. A slight decrease in the total length (mean = 31.0 mm) of the metamorph was noticeable (Table 1). Over the next few days, the reticulated color pattern of the limbs began to form and fully developed at stage 44 (Fig. 6B, C). The metamorphosing frog showed anatomical changes mainly in the mouth, where the angle was now located beneath the eye, in lateral view, and in tail reduction (Fig. 6D), beginning with the resorption of dorsal and ventral fins. Complete metamorphosis at stage 46 was reached after 79–91 days after observations began. During the transition to a juvenile, the total length decreased from a mean of 31.0 mm including the tail at stage 42 to a mean of 11.5 mm for the complete metamorph (stage 46), after complete reabsorption of the tail (Table 1).

## ﻿Discussion

Our results represent the first embryonic and larval staging for *R.variabilis*, providing detailed information on their initial life phases. These data are useful for future comparative studies between different populations of *R.variabilis*, and my help resolve biogeographic and taxonomic issues. Our data may also be useful for identify *R.variabilis* specimens at early life stages in the natural environment. The observations of captive-breeding contribute with knowledge to captive-breeding programs for the purpose of conserving endangered amphibian species. Nevertheless, the mortality rate (54%) suggests that the captive-breeding protocol needs improvement. The strong correlation between development measured by TL with that measured by photographically determined surface area of the silhouette of the individual suggests that image-based measurements are well suited for the analysis of development.

### ﻿Captive management and breeding

The captive-breeding protocol of Klein et al. (2020) that we followed for *R.variabilis* proved to be successful. In total 46% of the embryos reached metamorphosis. Although no average mortality rates are published for Dendrobatidae, we consider this a satisfactory result because a death rate of up to 80% has been estimated for tadpoles of other taxa bred in captivity (McWilliams 2008; Lisboa et al. 2021; McFadden et al. 2022). We made some casual observations of adult behavior, that although no quantification method was applied, the results can be used as generalizations and preliminary notes for future studies. The observation that adults assembled into groups prior to oviposition coincides with the promiscuous mating system behavior reported for the species group (Brown et al. 2011; Muell et al. 2022). The apparent preference from *R.variabilis* to lay eggs directly above the edge of the water is consistent with observations in the natural environment in French Guiana (Poelman and Dicke 2007). By contrast, Masche et al. (2010) reported for a *R.variabilis* population from Peru that eggs were laid under the water surface within phytotelmata, reinforcing the given need for further studies comparing eastern and western populations of *R.variabilis*. The egg deposition was observed more frequently in largest and most basal axils of *V.splendens* bromeliads that in the film containers. When detected under the water on the bottom of the film containers, the clutches were stepped on by adult frogs, and sometimes adults even defecated inside the containers on top of the spawn. However, probably as the adult individuals stepped on the eggs, they ended up slipping to the bottom of the film containers, rather than remaining above the water as in the bromeliads. Despite film containers being suitable for egg deposition in other *Ranitomeya* species (Klein et al. 2020), we feel the use of film containers for captive breeding of *R.variabilis* should be re-evaluated, and suggest the use of natural phytotelmata such as bromeliads instead.

Four tadpoles of *R.variabilis* showed malformations and died before completing metamorphosis. Similar results were reported for captive-bred tadpoles of *D.quinquevittatus*, a synonym of *R.variabilis*, where some tadpoles showed paralyzed or atrophied forelimbs, leading to their death (Lescure and Bechter 1982). A fifth individual studied here developed to the stage 41, had a smaller size than the others and died before completing metamorphosis. A similar outcome for tadpoles of the Reticulated Poison Frog (*R.reticulata*; *n* = 5) is reported in Klein et al. (2020). In this study, all tadpoles of *R.reticulata* had a smaller mean size and had more time to reach stage 41 compared to normal developed individuals. Not a single specimen out of five *R.reticulata* completed the metamorphosis and all died at 63 days, which was not the case in all individuals from that study from Klein et al (2020).

In a review assessing tadpole survival rates in experimental sets, Melvin and Houlahan (2012) indicated that the expected survival rate of laboratory bred tadpoles is generally higher compared to natural populations. Different theories about the mortality of tadpoles bred in captivity were formulated. For example, weaker individuals that would not survive in natural environments are able to survive in captivity, and these may generate weaker offspring with a lower chance of survival (Melvin and Houlahan 2012). Alternatively, death rates may be increased due to the offered artificial food, which may be different from the natural diet. Or, it may be related to the proliferation of lethal bacteria or fungi resulting from food decomposition (McWilliams 2008). More recently, deaths of larvae and metamorphs have been linked to Spiny Leg Syndrome, a common anomaly of musculoskeletal origin associated with captive breeding of amphibians (Camperio Ciani et al. 2018; Lassiter et al. 2020). The factors that trigger Spiny Leg Syndrome are still not entirely clear, but studies indicate that factors such as water quality, lack or excess nutrients and minerals are related to this anomaly (Barrows et al. 2017; Camperio Ciani et al. 2018; Lassiter et al. 2020). Given our small sample size, we assume that it may be premature to infer that the high mortality in our sample is integrally related to this syndrome. But we consider that the malformations, which developed in specimens at the metamorph stages, are a characteristic manifestation of this Spiny Leg Syndrome, and so we assume that tanking a note would be valid.

### ﻿Growth and development

For *R.variabilis*, complete metamorphosis time took an average of 81.8 days at 24 °C. These data are slightly different from that observed for the sister specie *R.amazonica*. Where, under the same conditions of captive breeding, at 24 °C and with the same type food resource, complete metamorphosis time took an average of 96 days (Klein et al. 2020).

The characteristics of developmental stages described for *R.variabilis* largely coincide with those for the generalized staging system of Gosner (1960). We observed only two morphological differences. First, the labia and teeth differentiation occurred in stage 24, one stage later than indicated in the general staging table. Second, atrophy of the oral apparatus began in stage 40, one stage earlier than determined in the generalized system for two of the six tadpoles studied at this stage. Deviations from the generalized staging system have also been observed in other studies for other captive-bred *Ranitomeya* species. Where, for spotted poison frog (*R.vanzolinii* (Myers, 1982)) the atrophy of the buccal apparatus occurred later than expected by the Gosner staging system, and for *R.amazonica* atrophy of the ventral tube occurred later than expected (Klein et al. 2020).

The TL of *R.variabilis* tadpoles, arise from adult captive-bred specimens from French Guiana, is a possible distinctive morphological characteristic for the individuals of this population. Due to the fact that, the herein studied tadpoles present a larger TL, in comparison with the individuals of *R.variabilis* of Peru, ecological field data (Masche et al. 2010). Were, the TL for a Peruvian tadpole of *R.variabilis* (field data) at stages 25 and 41 was 10.5 and 17.9 mm (*n* = 1), whereas the TL of French Guiana tadpoles (captive breed) presented a TL range of 11.2–18 and 29.0–34.0 mm (*n* = 6 or 7), respectively. In comparison with tadpoles of the sister specie raised under the same captive breeding conditions, the TL of *R.variabilis* (*n* = 6) in development stage 26 is 17.8 mm, were *R.amazonica* (*n* = 5), had a TL 15.5 mm in the same stage (Klein et al. 2020). Additional TL comparisons between *R.amazonica* and *R.variabilis* breed in captivity tadpoles were respectively: stage 31, 20.9 and 25.6 mm; stage 34, 23.5 and 28.7 mm; and stage 41, 27.7 and 31.7 mm. In addition, TL data from tadpoles of other *Ranitomeya* species also exhibit a smaller size in comparison with *R.variabilis* tadpoles described here (see von May et al. 2008; Brown et al. 2011; Klein et al. 2020), which suggests that a higher TL could be a specific morphological feature of French Guiana *R.variabilis* tadpoles, and may help to facilitate the identification of tadpoles of *R.variabilis* in the wild. Moreover, this morphological feature may serve as a hint to uncover cryptic species within the putative species complex *R.variabilis* (Brown et al. 2011; Muell et al. 2022). A caveat, however, is that we raised the tadpoles under artificial conditions, including a lack of food limitations, constant temperatures, and without competition. These were therefore very different from natural conditions. Thus, a comparison with tadpoles obtained from natural environments would be indispensable and helpful in evaluating whether the species size differences are real.

**Figure 7.**
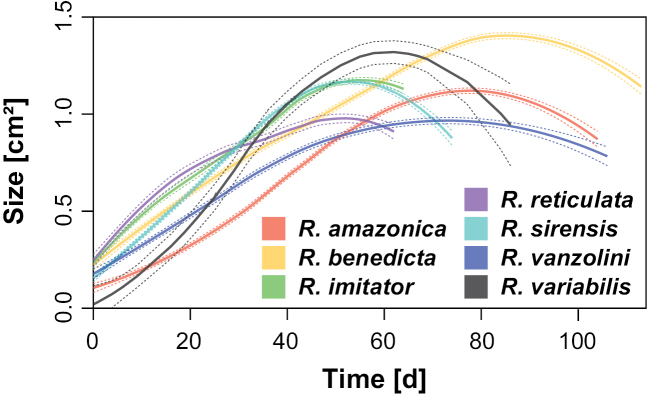
Comparison of growth curve of *Ranitomeyavariabilis* from French Guiana (from Fig. 1), with growth curves for *R.amazonica*, *R.benedicta*, *R.imitator*, *R.reticulata*, *R.sirensis*, and *R.vanzolinii*, data adapted from Klein et al. (2020), from embryo to metamorph. Size is represented by surface area of silhouette of individual. Solid lines represent the results from the local polynomial regression (Loess); dotted lines show the respective 95% confidence intervals.

The SAISAQ method for measuring individual length and surface area of the silhouette and the staging as a growth evaluation method allowed us to compile a complete development dataset. The growth curve of the tadpoles reflects the development of embryo to metamorph and the relationship between body mass as estimated by surface area and length. Where, through the generated curve, the body size increase is evidenced throughout its development, as well as the decrease in body proportion in the final stages of development. We also present here the results of SAISAQ for the developmental stages 42 and 43, which is different from the recommendations in Kurth et al. (2014) as the developing limbs can influence the results and distort the relationship between the dorsal surface area of silhouette and the mass of the individuals. However, although the results obtained after stage 41 may be less accurate, our results for these two stages still generate an expected growth curve (Figs 1, 7). Where, the relationship between the dorsal surface size and the mass of the individuals decreases in the last metamorphosis stages, as expected for tadpoles.

A comparison of the growth curves for seven *Ranitomeya* species in early life stages based on surface area of the silhouette (Klein et al. 2020), highlights the difference in development time and size of tadpoles of *R.variabilis* (Fig. 7). The peak size for *R.variabilis* tadpoles was larger than that for five other species, including the sister species *R.amazonica*. Only the Blessed Poison Frog (*R.benedicta* Brown, Twomey, Pepper, and Sanchez-Rodriguez, 2008) tadpoles were larger than *R.variabilis* at peak size. Moreover, the total development time is different in *R.benedicta*, which needs the largest development time to complete metamorphosis (Table 2), and also has the highest tadpole body proportions. Although the tadpoles of *R.amazonica* are smaller than those of *R.variabilis*, which is its sister species, the development time in captivity under the same husbandry conditions was seven days longer (up to 99 days; Fig. 7, Klein et al. 2020). Our data can help to increase the knowledge about the reproductive biology of this species and may be useful for *ex situ* breeding programs for conservation purposes.

**Table 2.** Comparison of developmental time for *Ranitomeyavariabilis* from French Guiana, with developmental time for *R.amazonica* (Amazonian poison dart frog), *R.benedicta* (Blessed poison frog), *R.imitator* (Mimic poison frog), *R.reticulata* (Reticulated poison frog), *R.sirensis* (Sira poison frog), and *R.vanzolinii* (Spotted poisonfFrog); data adapted from Klein et al. (2020). Stage = Gosner stage of development; grouped numbers represent mean (range).

Stage	*R.amazonica*	*R.benedicta*	*R.imitator*	*R.reticulata*	*R.sirensis*	*R.vanzolinii*	*R.variabilis*
8	1	–	1	–	–	–	1
9	–	–	2	–	–	–	–
10	2	–		–	–	–	–
11	–	–	3	–	–	–	2
13	3	–		–	–	–	–
14	–	–	4	–	–	–	–
19	5	–	5	–	–	–	5
20	6	–	6	–	–	–	6
21	7	–	7	–	–	–	–
22	8	–	8	–	–	–	8
23	9	–	10	–	–	–	–
24	10	–	11	–	–	–	11
25	11	–	14	–	–	–	16.8 (11–22)
28	56 (49–67)	61 (54–70)	34 (24–43)	36 (29–41)	31 (24–39)	32–52	27.0 (26–29)
41	84 (69–88)	88 (80–94)	51 (48–53)	56 (42–63)	52 (47–56)	60 (51–73)	64.5 (59–69)
42	89 (82–94)	105	63	–	60 (56–65)	73 (64–94)	68.5 (68–71)
43	–	112	–	–	–	–	70.7 (70–73)
44–46	96 (84–105)	114	67	–	63 (60–71)	77 (61–107)	82.4 (80–92)

## ﻿Additional information

### Author ORCIDs

Ruth Anastasia Regnet https://orcid.org/0000-0001-6929-4084

Inna Rech https://orcid.org/0009-0000-2356-4405

Dennis Rödder https://orcid.org/0000-0002-6108-1639

Mirco Solé https://orcid.org/0000-0001-7881-6227

### Data availability

All of the data that support the findings of this study are available in the main text or Supplementary Information.
